# Supplementary material for: Designing Optimal Breakfast for the United States Using Linear Programming and the NHANES 2011–2014 Database: A Study from the International Breakfast Research Initiative (IBRI)
Source: Nutrients. 2019 Jun 19;11(6):1374. doi: 10.3390/nu11061374 (PMC6627424; doi:10.3390/nu11061374)
Supplement: Supplementary file 1 [file nutrients-11-01374-s001.pdf]

Table S1. Quantity consumed (grams) and contribution (%) of WWEIA food categories to MyPlate food groups in Adults observed diets.

		OBS quantity	Other Fruit	Citrus Fruit	Juice	Whole grains	Ref grains	Soy, nuts	Eggs	Meat	Cheese	Yogurt	Milk
Beverages	Coffee & Tea	231.4	0.00	0.00	0.00	0.01	0.00	0.00	0.00	0.00	0.00	0.00	10.78
	Diet Beverages	12.4	0.00	0.00	0.04	0.00	0.00	0.00	0.00	0.00	0.00	0.00	0.00
	Sweetened Beverages	75.5	22.11	42.47	23.26	0.12	0.07	2.69	0.00	0.00	0.00	19.62	5.56
Fats & Oils	Fats & Oils	9.1	0.00	0.00	0.00	0.00	0.00	0.00	0.08	0.00	0.01	0.04	1.97
Fruit	Fruit	9.3	62.66	45.23	0.07	0.00	0.00	0.00	0.00	0.00	0.00	0.00	0.00
	100% Juice	15.8	0.00	0.00	74.99	0.00	0.00	0.00	0.00	0.00	0.00	0.00	0.00
Grains	Breads	16.2	1.65	0.00	0.00	49.29	32.30	1.49	0.00	0.00	0.21	0.01	0.20
	Cooked grains	8.4	0.05	0.00	0.00	13.51	0.56	0.01	0.01	0.00	0.29	0.00	2.80
	Grains	1.3	0.28	0.34	0.00	1.40	0.70	0.00	0.01	0.00	0.00	0.00	0.01
	Quick Breads	11.6	0.44	4.42	0.04	3.95	17.23	1.36	5.68	0.00	0.04	0.00	4.76
	High Sugar RTE Cereal	3.3	3.52	0.10	0.00	12.98	1.90	2.46	0.00	0.00	0.00	0.00	0.15
	Low Sugar RTE Cereal	1.7	0.12	0.00	0.00	10.43	1.05	2.00	0.00	0.00	0.00	0.00	0.00
Milk & Dairy	Cheese	2.2	0.02	0.00	0.00	0.00	0.02	0.00	0.00	0.00	41.11	0.00	0.00
	Flavored Milk	4.5	0.00	0.00	0.00	0.00	0.00	0.00	0.00	0.00	0.00	0.00	6.20
	Milk	32.8	0.00	0.00	0.00	0.00	0.00	0.00	0.00	0.00	0.00	0.00	55.56
	Milk Dessert Drinks	0.6	0.00	0.00	0.00	0.00	0.00	0.25	0.00	0.00	0.00	0.00	0.45
	Yogurt	4.5	0.51	2.43	0.00	0.00	0.00	0.00	0.00	0.00	0.00	76.75	0.00
Mixed Dishes	Mixed Dishes	33.7	0.20	0.01	0.06	1.66	24.08	17.26	13.18	23.80	46.55	0.04	3.41
Protein Foods	Eggs	21.7	0.00	0.00	0.00	0.00	0.07	0.00	77.82	4.84	11.04	0.00	4.50
	Nuts, Beans & Soy	1.8	0.24	0.00	0.00	0.23	0.01	55.94	0.02	0.03	0.00	0.00	0.00
	Processed Meat	9.1	0.00	0.00	0.00	0.00	0.00	0.01	0.00	46.42	0.03	0.00	0.00
	Seafood/Meat	4.9	0.00	0.00	0.00	0.01	1.01	0.20	0.41	24.87	0.00	0.00	0.07
	Candy	0.3	0.09	0.00	0.00	0.00	0.01	3.18	0.00	0.00	0.00	0.00	0.10

Snacks & Sweets	Crackers	0.4	0.00	0.00	0.00	0.69	1.09	0.80	0.00	0.00	0.03	0.00	0.00
	Other Desserts	1.0	0.11	0.00	0.02	0.00	0.02	0.09	0.01	0.00	0.01	3.51	0.78
	Savory Snacks	0.7	0.00	0.00	0.00	1.66	1.29	0.09	0.00	0.00	0.09	0.00	0.00
	Snack/Meal Bars	1.0	0.30	0.08	0.00	2.77	0.36	7.38	0.00	0.00	0.00	0.03	0.12
	Sweet Bakery	12.8	6.55	0.79	0.01	1.29	18.22	4.35	2.61	0.00	0.07	0.00	2.34
Sugars	Sugars	8.6	1.15	4.12	1.51	0.00	0.00	0.41	0.00	0.00	0.00	0.00	0.10
Vegetables	Vegetables, Non-potato	2.2	0.00	0.00	0.00	0.00	0.01	0.02	0.01	0.00	0.09	0.01	0.01
	White Potatoes	8.7	0.00	0.00	0.00	0.00	0.01	0.00	0.15	0.04	0.44	0.00	0.14

Table S2: Optimized quantity (grams) and contribution (%) of WWEIA food categories to MyPlate food groups in Adults optimized diets (relative function)

		OPT quantity	Other Fruit	Citrus Fruit	Juice	Whole grains	Ref grains	Soy, nuts	Eggs	Meat	Chees e	Yogurt	Milk
Beverages	Coffee & Tea	231.4	0.00	0.00	0.00	0.01	0.00	0.00	0.00	0.00	0.00	0.00	2.59
	Diet Beverages	12.4	0.00	0.00	0.04	0.00	0.00	0.00	0.00	0.00	0.00	0.00	0.00
	Sweetened Beverages	75.5	3.30	8.55	23.14	0.07	0.12	2.91	0.00	0.00	0.00	19.63	1.34
Fats & Oils	Fats & Oils	9.1	0.00	0.00	0.00	0.00	0.00	0.00	6.17	0.00	0.01	0.04	0.47
Fruit	Fruit	92.1	92.21	89.88	0.70	0.00	0.00	0.00	0.00	0.00	0.00	0.00	0.00
	100% Juice	15.8	0.00	0.00	74.60	0.00	0.00	0.00	0.00	0.00	0.00	0.00	0.00
Grains	Breads	16.2	0.25	0.00	0.00	28.98	58.51	1.61	0.38	0.00	0.50	0.01	0.05
	Cooked grains	8.4	0.01	0.00	0.00	7.94	1.01	0.01	1.14	0.00	0.68	0.00	0.67
	Grains	1.3	0.04	0.07	0.00	0.82	1.27	0.00	0.62	0.00	0.00	0.00	0.00
	Quick Breads	0	0.00	0.00	0.00	0.00	0.00	0.00	0.00	0.00	0.00	0.00	0.00
	High Sugar RTE Cereal	22.9	3.63	0.14	0.00	52.75	23.76	18.37	0.00	0.00	0.00	0.00	0.25
	Low Sugar RTE Cereal	1.7	0.02	0.00	0.00	6.13	1.90	2.16	0.00	0.00	0.00	0.00	0.00
Milk & Dairy	Cheese	2.2	0.00	0.00	0.00	0.00	0.03	0.00	0.00	0.00	97.22	0.00	0.00
	Flavored Milk	4.5	0.00	0.00	0.00	0.00	0.00	0.00	0.00	0.00	0.00	0.00	1.49
	Milk	227.6	0.00	0.00	0.00	0.00	0.00	0.00	0.00	0.00	0.00	0.00	92.61
	Milk Dessert Drinks	0.6	0.00	0.00	0.00	0.00	0.00	0.27	0.03	0.00	0.00	0.00	0.11
	Yogurt	4.5	0.08	0.49	0.00	0.00	0.00	0.00	0.00	0.00	0.00	76.78	0.00
Mixed Dishes	Mixed Dishes	0	0.00	0.00	0.00	0.00	0.00	0.00	0.00	0.00	0.00	0.00	0.00
Protein Foods	Eggs	0	0.00	0.00	0.00	0.00	0.00	0.00	0.00	0.00	0.00	0.00	0.00
	Nuts, Beans & Soy	1.8	0.04	0.00	0.00	0.14	0.01	60.55	1.91	0.12	0.01	0.00	0.00
	Processed Meat	0	0.00	0.00	0.00	0.00	0.00	0.00	0.00	0.00	0.00	0.00	0.00
	Seafood/Meat	4.9	0.00	0.00	0.00	0.00	1.84	0.22	33.48	99.72	0.00	0.00	0.02

Snacks & Sweets	Candy	0.3	0.01	0.00	0.00	0.00	0.02	3.45	0.04	0.00	0.00	0.00	0.02
	Crackers	0.4	0.00	0.00	0.00	0.41	1.97	0.87	0.00	0.00	0.07	0.00	0.00
	Other Desserts	1.0	0.02	0.00	0.02	0.00	0.04	0.10	1.04	0.00	0.03	3.51	0.19
	Savory Snacks	0.7	0.00	0.00	0.00	0.97	2.33	0.10	0.00	0.00	0.21	0.00	0.00
	Snack/Meal Bars	1.0	0.05	0.02	0.00	1.63	0.65	7.99	0.16	0.00	0.00	0.03	0.03
	Sweet Bakery	2.5	0.19	0.03	0.00	0.15	6.49	0.93	41.93	0.00	0.03	0.00	0.11
Sugars	Sugars	8.6	0.17	0.83	1.50	0.00	0.00	0.45	0.00	0.00	0.00	0.00	0.02
Vegetables	Vegetables, Non-potato	2.2	0.00	0.00	0.00	0.00	0.02	0.02	0.88	0.00	0.21	0.01	0.00
	White Potatoes	8.7	0.00	0.00	0.00	0.00	0.03	0.00	12.21	0.16	1.03	0.00	0.03

Table S3: Optimized quantity (grams) and contribution (%) of WWEIA food categories to MyPlate food groups in Adults optimized diets (absolute function)

		OPT quantity	Other Fruit	Citrus Fruit	Juice	Whole grains	Ref grains	Soy, nuts	Eggs	Meat	Chees e	Yogurt	Milk
Beverages	Coffee & Tea	231.4	0.00	0.00	0.00	0.00	0.00	0.00	0.00	0.00	0.00	0.00	2.84
	Diet Beverages	12.4	0.00	0.00	0.04	0.00	0.00	0.00	0.00	0.00	0.00	0.00	0.00
	Sweetened Beverages	75.5	4.03	10.04	23.17	0.06	0.24	2.51	0.00	0.00	0.00	19.63	1.47
Fats & Oils	Fats & Oils	9.1	0.00	0.00	0.00	0.00	0.00	0.00	0.10	0.00	0.04	0.04	0.52
Fruit	Fruit	77.1	94.21	88.25	0.59	0.00	0.00	0.00	0.00	0.00	0.00	0.00	0.00
	100% Juice	15.8	0.00	0.00	74.68	0.00	0.00	0.00	0.00	0.00	0.00	0.00	0.00
Grains	Breads	0	0.00	0.00	0.00	0.00	0.00	0.00	0.00	0.00	0.00	0.00	0.00
	Cooked grains	8.4	0.01	0.00	0.00	6.49	2.01	0.01	0.02	0.00	2.40	0.00	0.74
	Grains	1.3	0.05	0.08	0.00	0.67	2.52	0.00	0.01	0.00	0.00	0.00	0.00
	Quick Breads	0	0.00	0.00	0.00	0.00	0.00	0.00	0.00	0.00	0.00	0.00	0.00
	High Sugar RTE Cereal	3.3	0.64	0.02	0.00	6.23	6.83	2.29	0.00	0.00	0.00	0.00	0.04
	Low Sugar RTE Cereal	27.9	0.36	0.00	0.00	84.16	63.34	31.29	0.00	0.00	0.00	0.00	0.00
Milk & Dairy	Cheese	0	0.00	0.00	0.00	0.00	0.00	0.00	0.00	0.00	0.00	0.00	0.00
	Flavored Milk	4.5	0.00	0.00	0.00	0.00	0.00	0.00	0.00	0.00	0.00	0.00	1.64
	Milk	203.6	0.00	0.00	0.00	0.00	0.00	0.00	0.00	0.00	0.00	0.00	90.96
	Milk Dessert Drinks	0.6	0.00	0.00	0.00	0.00	0.01	0.23	0.00	0.00	0.00	0.00	0.12
	Yogurt	4.5	0.09	0.57	0.00	0.00	0.00	0.00	0.00	0.00	0.00	76.79	0.00
Mixed Dishes	Mixed Dishes	0	0.00	0.00	0.00	0.00	0.00	0.00	0.00	0.00	0.00	0.00	0.00
Protein Foods	Eggs	21.7	0.00	0.00	0.00	0.00	0.26	0.00	98.35	16.26	92.17	0.00	1.19
	Nuts, Beans & Soy	1.8	0.04	0.00	0.00	0.11	0.03	52.14	0.03	0.10	0.04	0.00	0.00
	Processed Meat	0	0.00	0.00	0.00	0.00	0.00	0.00	0.00	0.00	0.00	0.00	0.00
	Seafood/Meat	4.9	0.00	0.00	0.00	0.00	3.65	0.19	0.52	83.51	0.00	0.00	0.02

Snacks & Sweets	Candy	0.3	0.02	0.00	0.00	0.00	0.04	2.97	0.00	0.00	0.00	0.00	0.03
	Crackers	0	0.00	0.00	0.00	0.00	0.00	0.00	0.00	0.00	0.00	0.00	0.00
	Other Desserts	1.0	0.02	0.00	0.02	0.00	0.08	0.09	0.02	0.00	0.11	3.51	0.20
	Savory Snacks	0.7	0.00	0.00	0.00	0.80	4.63	0.09	0.00	0.00	0.72	0.00	0.00
	Snack/Meal Bars	1.0	0.06	0.02	0.00	1.33	1.30	6.88	0.00	0.00	0.00	0.03	0.03
	Sweet Bakery	2.9	0.27	0.04	0.00	0.14	14.98	0.93	0.75	0.00	0.13	0.00	0.14
Sugars	Sugars	8.6	0.21	0.97	1.50	0.00	0.00	0.38	0.00	0.00	0.00	0.00	0.03
Vegetables	Vegetables, Non-potato	2.2	0.00	0.00	0.00	0.00	0.03	0.02	0.01	0.00	0.73	0.01	0.00
	White Potatoes	8.7	0.00	0.00	0.00	0.00	0.05	0.00	0.19	0.13	3.65	0.00	0.04

Table S4: Quantity consumed (grams) and contribution (%) of WWEIA food categories to MyPlate food groups in children observed diets

		OBS quantity	Other Fruit	Citrus Fruit	Juice	Whole grains	Ref grains	Soy, nuts	Eggs	Meat	Cheese	Yogurt	Milk
Beverages	Coffee & Tea	24.3	0.00	0.00	0.00	0.00	0.00	0.02	0.00	0.00	0.00	0.00	1.70
	Diet Beverages	2.6	0.00	0.00	0.00	0.00	0.00	0.00	0.00	0.00	0.00	0.00	0.00
	Sweetened Beverages	55.9	13.38	38.38	14.35	0.00	0.03	1.19	0.00	0.00	0.00	29.16	1.25
Fats & Oils	Fats & Oils	1.7	0.00	0.00	0.00	0.00	0.00	0.00	0.05	0.00	0.00	0.01	0.01
Fruit	Fruit	8.9	69.84	48.62	0.29	0.00	0.00	0.00	0.00	0.00	0.00	0.00	0.00
	100% Juice	19.5	0.00	0.00	84.44	0.00	0.00	0.00	0.00	0.00	0.00	0.00	0.00
Grains	Breads	7.3	0.61	0.00	0.00	26.85	13.58	1.62	0.03	0.00	0.17	0.00	0.09
	Cooked grains	6.9	0.00	0.00	0.00	13.63	0.49	0.00	0.09	0.00	2.06	0.00	1.73
	Grains	0.9	0.00	0.00	0.00	0.03	0.64	0.50	0.03	0.00	0.00	0.00	0.00
	Quick Breads	20.0	0.09	9.33	0.10	10.82	26.05	0.95	17.47	0.00	0.00	0.00	4.88
	High Sugar RTE Cereal	6.4	1.76	0.05	0.00	28.31	3.88	4.65	0.00	0.00	0.00	0.00	0.07
	Low Sugar RTE Cereal	0.8	0.00	0.00	0.00	6.22	0.61	0.72	0.00	0.00	0.00	0.00	0.00
Milk & Dairy	Cheese	0.8	0.00	0.00	0.00	0.00	0.00	0.00	0.00	0.00	25.64	0.00	0.00
	Flavored Milk	9.7	0.00	0.00	0.00	0.00	0.00	0.00	0.00	0.00	0.00	0.00	8.92
	Milk	75.1	0.00	0.00	0.00	0.00	0.00	0.00	0.00	0.00	0.00	0.00	73.98
	Milk Dessert Drinks	0.8	0.00	0.00	0.00	0.00	0.00	0.55	0.01	0.00	0.00	0.00	0.45
	Yogurt	2.7	0.50	1.68	0.00	0.00	0.00	0.00	0.00	0.00	0.00	66.86	0.00
Mixed Dishes	Mixed Dishes	26.8	0.11	0.00	0.05	7.20	21.79	27.68	10.55	32.61	60.66	0.00	1.37
Protein Foods	Eggs	12.9	0.00	0.00	0.00	0.00	0.02	0.00	67.95	4.34	10.57	0.00	2.22
	Nuts, Beans & Soy	0.5	0.32	0.00	0.00	0.00	0.01	39.19	0.02	0.00	0.00	0.00	0.00
	Processed Meat	6.4	0.00	0.00	0.00	0.00	0.00	0.00	0.00	47.37	0.00	0.00	0.00
	Seafood/Meat	2.2	0.00	0.00	0.00	0.13	0.57	0.07	0.25	15.68	0.00	0.00	0.02
Snacks & Sweets	Candy	0.6	1.87	0.00	0.00	0.00	0.00	4.81	0.00	0.00	0.00	0.00	0.18
	Crackers	0.5	0.00	0.00	0.00	2.03	0.96	2.32	0.00	0.00	0.03	0.00	0.00
	Other Desserts	0.3	0.06	0.00	0.00	0.00	0.02	0.00	0.02	0.00	0.00	3.96	0.07
	Savory Snacks	0.9	0.00	0.00	0.00	2.03	1.41	0.00	0.00	0.00	0.10	0.00	0.00

	Snack/Meal Bars	0.4	0.07	0.04	0.00	1.65	0.15	5.01	0.00	0.00	0.00	0.00	0.06
	Sweet Bakery	20.6	11.19	1.07	0.00	1.10	29.80	7.91	3.50	0.00	0.78	0.00	2.88
Sugars	Sugars	7.5	0.20	0.84	0.76	0.00	0.00	2.79	0.00	0.00	0.00	0.00	0.13
Vegetables	Vegetables, Non-potato	0.4	0.00	0.00	0.00	0.00	0.00	0.01	0.01	0.00	0.00	0.01	0.00
	White Potatoes	1.7	0.00	0.00	0.00	0.00	0.00	0.00	0.00	0.00	0.00	0.00	0.00

Table S5: Optimized quantity (grams) and contribution (%) of WWEIA food categories to MyPlate food groups in children optimized diets (relative function)

		OPT quantity	Other Fruit	Citrus Fruit	Juice	Whole grains	Ref grains	Soy, nuts	Eggs	Meat	Chees e	Yogurt	Milk
Beverages	Coffee & Tea	24.3	0.00	0.00	0.00	0.00	0.00	0.02	0.00	0.00	0.00	0.00	0.56
	Diet Beverages	2.6	0.00	0.00	0.00	0.00	0.00	0.00	0.00	0.00	0.00	0.00	0.00
	Sweetened Beverages	55.9	1.14	4.54	13.74	0.00	0.05	1.52	0.00	0.00	0.00	29.16	0.41
Fats & Oils	Fats & Oils	1.7	0.00	0.00	0.00	0.00	0.00	0.00	0.39	0.00	0.01	0.01	0.00
Fruit	Fruit	145.7	97.84	94.36	4.59	0.00	0.00	0.00	0.00	0.00	0.00	0.00	0.00
	100% Juice	19.5	0.00	0.00	80.87	0.00	0.00	0.00	0.00	0.00	0.00	0.00	0.00
Grains	Breads	7.3	0.05	0.00	0.00	16.04	24.58	2.07	0.23	0.00	0.60	0.00	0.03
	Cooked grains	6.9	0.00	0.00	0.00	8.14	0.88	0.00	0.71	0.00	7.32	0.00	0.57
	Grains	0.9	0.00	0.00	0.00	0.02	1.16	0.64	0.25	0.00	0.00	0.00	0.00
	Quick Breads	13.7	0.01	0.76	0.07	4.43	32.35	0.83	92.20	0.00	0.00	0.00	1.11
	High Sugar RTE Cereal	24.2	0.57	0.02	0.00	64.08	26.60	22.55	0.00	0.00	0.00	0.00	0.09
	Low Sugar RTE Cereal	0.8	0.00	0.00	0.00	3.72	1.10	0.93	0.00	0.00	0.00	0.00	0.00
Milk & Dairy	Cheese	0.8	0.00	0.00	0.00	0.00	0.00	0.00	0.00	0.00	91.24	0.00	0.00
	Flavored Milk	9.7	0.00	0.00	0.00	0.00	0.00	0.00	0.00	0.00	0.00	0.00	2.95
	Milk	288.0	0.00	0.00	0.00	0.00	0.00	0.00	0.00	0.00	0.00	0.00	93.84
	Milk Dessert Drinks	0.8	0.00	0.00	0.00	0.00	0.00	0.71	0.09	0.00	0.00	0.00	0.15
	Yogurt	2.7	0.04	0.20	0.00	0.00	0.00	0.00	0.00	0.00	0.00	66.86	0.00
Mixed Dishes	Mixed Dishes	0	0.00	0.00	0.00	0.00	0.00	0.00	0.00	0.00	0.00	0.00	0.00
Protein Foods	Eggs	0	0.00	0.00	0.00	0.00	0.00	0.00	0.00	0.00	0.00	0.00	0.00
	Nuts, Beans & Soy	0.5	0.03	0.00	0.00	0.00	0.01	50.11	0.19	0.01	0.00	0.00	0.00
	Processed Meat	0	0.00	0.00	0.00	0.00	0.00	0.00	0.00	0.00	0.00	0.00	0.00
	Seafood/Meat	2.2	0.00	0.00	0.00	0.08	1.03	0.09	1.93	99.99	0.00	0.00	0.01

Snacks & Sweets	Candy	0.6	0.16	0.00	0.00	0.00	0.00	6.16	0.00	0.00	0.00	0.00	0.06
	Crackers	0.5	0.00	0.00	0.00	1.21	1.75	2.96	0.00	0.00	0.09	0.00	0.00
	Other Desserts	0.3	0.00	0.00	0.00	0.00	0.03	0.00	0.12	0.00	0.00	3.96	0.02
	Savory Snacks	0.9	0.00	0.00	0.00	1.21	2.55	0.00	0.00	0.00	0.34	0.00	0.00
	Snack/Meal Bars	0.4	0.01	0.00	0.00	0.99	0.27	6.40	0.00	0.00	0.00	0.00	0.02
	Sweet Bakery	2.9	0.14	0.02	0.00	0.09	7.63	1.43	3.81	0.00	0.39	0.00	0.13
Sugars	Sugars	7.5	0.02	0.10	0.73	0.00	0.00	3.57	0.00	0.00	0.00	0.00	0.04
Vegetables	Vegetables, Non-potato	0.4	0.00	0.00	0.00	0.00	0.00	0.01	0.09	0.00	0.00	0.01	0.00
	White Potatoes	1.7	0.00	0.00	0.00	0.00	0.00	0.00	0.00	0.00	0.00	0.00	0.00

Table S6: Optimized quantity (grams) and contribution (%) of WWEIA food categories to MyPlate food groups in children optimized diets (absolute function)

		OPT quantity	Other Fruit	Citrus Fruit	Juice	Whole grains	Ref grains	Soy, nuts	Eggs	Meat	Chees e	Yogurt	Milk
Beverages	Coffee & Tea	24.3	0.00	0.00	0.00	0.00	0.00	0.00	0.00	0.00	0.00	0.00	0.66
	Diet Beverages	2.6	0.00	0.00	0.00	0.00	0.00	0.00	0.00	0.00	0.00	0.00	0.00
	Sweetened Beverages	55.9	1.41	5.67	13.88	0.00	0.09	0.06	0.00	0.00	0.00	29.16	0.49
Fats & Oils	Fats & Oils	0	0.00	0.00	0.00	0.00	0.00	0.00	0.00	0.00	0.00	0.00	0.00
Fruit	Fruit	116.2	96.41	93.94	3.70	0.00	0.00	0.00	0.00	0.00	0.00	0.00	0.00
	100% Juice	19.5	0.00	0.00	81.68	0.00	0.00	0.00	0.00	0.00	0.00	0.00	0.00
Grains	Breads	7.3	0.06	0.00	0.00	17.16	41.16	0.09	0.04	0.00	0.80	0.00	0.03
	Cooked grains	6.9	0.00	0.00	0.00	8.71	1.47	0.00	0.13	0.00	9.77	0.00	0.67
	Grains	0.9	0.00	0.00	0.00	0.02	1.95	0.03	0.05	0.00	0.00	0.00	0.00
	Quick Breads	0	0.00	0.00	0.00	0.00	0.00	0.00	0.00	0.00	0.00	0.00	0.00
	High Sugar RTE Cereal	6.4	0.19	0.01	0.00	18.09	11.75	0.25	0.00	0.00	0.00	0.00	0.03
	Low Sugar RTE Cereal	11.2	0.00	0.00	0.00	52.95	24.61	0.51	0.00	0.00	0.00	0.00	0.00
Milk & Dairy	Cheese	0	0.00	0.00	0.00	0.00	0.00	0.00	0.00	0.00	0.00	0.00	0.00
	Flavored Milk	9.7	0.00	0.00	0.00	0.00	0.00	0.00	0.00	0.00	0.00	0.00	3.47
	Milk	243.6	0.00	0.00	0.00	0.00	0.00	0.00	0.00	0.00	0.00	0.00	93.33
	Milk Dessert Drinks	0.8	0.00	0.00	0.00	0.00	0.01	0.03	0.02	0.00	0.00	0.00	0.17
	Yogurt	2.7	0.05	0.25	0.00	0.00	0.00	0.00	0.00	0.00	0.00	66.87	0.00
Mixed Dishes	Mixed Dishes	3.6	0.00	0.00	0.01	0.62	8.87	0.20	2.00	17.88	38.68	0.00	0.07
Protein Foods	Eggs	12.9	0.00	0.00	0.00	0.00	0.07	0.00	95.62	17.71	50.19	0.00	0.86
	Nuts, Beans & Soy	25.0	1.60	0.00	0.00	0.00	0.74	98.14	1.60	0.40	0.00	0.00	0.00
	Processed Meat	0	0.00	0.00	0.00	0.00	0.00	0.00	0.00	0.00	0.00	0.00	0.00
	Seafood/Meat	2.2	0.00	0.00	0.00	0.08	1.73	0.00	0.35	64.01	0.00	0.00	0.01

Snacks & Sweets	Candy	0.6	0.20	0.00	0.00	0.00	0.01	0.26	0.00	0.00	0.00	0.00	0.07
	Crackers	0	0.00	0.00	0.00	0.00	0.00	0.00	0.00	0.00	0.00	0.00	0.00
	Other Desserts	0.3	0.01	0.00	0.00	0.00	0.05	0.00	0.02	0.00	0.00	3.96	0.03
	Savory Snacks	0.9	0.00	0.00	0.00	1.30	4.27	0.00	0.00	0.00	0.45	0.00	0.00
	Snack/Meal Bars	0.4	0.01	0.01	0.00	1.05	0.45	0.27	0.00	0.00	0.00	0.00	0.02
	Sweet Bakery	0.6	0.04	0.00	0.00	0.02	2.79	0.01	0.15	0.00	0.11	0.00	0.03
Sugars	Sugars	7.5	0.02	0.12	0.74	0.00	0.00	0.15	0.00	0.00	0.00	0.00	0.05
Vegetables	Vegetables, Non-potato	0.4	0.00	0.00	0.00	0.00	0.00	0.00	0.02	0.00	0.00	0.01	0.00
	White Potatoes	1.7	0.00	0.00	0.00	0.00	0.00	0.00	0.00	0.00	0.00	0.00	0.00
